# Parameters affecting the reliable measurement of fluconazole tolerance using disk diffusion assays with *Candida albicans*

**DOI:** 10.1128/spectrum.01390-26

**Published:** 2026-08-21

**Authors:** Nora Kawar, Judith Berman

**Affiliations:** 1 Shmunis School of Biomedicine and Cancer Research, George S. Wise Faculty of Life Sciences, Tel Aviv University 26745, Ramat Aviv, Israel; Stony Brook University, Stony Brook, New York, USA

**Keywords:** drug resistance, drug tolerance, antifungal diagnostics, antifungal drugs

## Abstract

Disk diffusion assays (DDAs) are widely used to determine antimicrobial susceptibility, which is measured as the size of the zone of inhibition (ZOI) after 24 h of growth, and is inversely proportional to the minimal inhibitory concentration (MIC). DDAs can also be used to measure the level of antifungal drug tolerance, defined as the degree to which a subpopulation of cells grows slowly at drug concentrations above the MIC. Tolerance occurs via one or more mechanisms that involve stress responses and that are distinguishable from mechanisms of drug resistance. Measurements of antifungal drug tolerance are generally more sensitive to growth parameters, such as temperature and pH. Here, we investigated parameters used for executing DDAs and asked if they affect the reproducibility of fluconazole susceptibility and tolerance measurements using *Candida albicans* isolates. Specifically, we tested drug format, media composition, and inoculum parameters. Drug format had little effect on DDA measurements, whereas media composition did affect assay outcomes. In general, inoculum density affected the ability to distinguish between the levels of drug susceptibility and tolerance in different strains. Of note, fluconazole tolerance measurements were much more sensitive than susceptibility measurements to the medium properties. Finally, we recommend conditions that accurately and reproducibly measure fluconazole susceptibility and tolerance levels in *C. albicans* isolates.

**IMPORTANCE:**

Disk diffusion assays are a popular method for testing antimicrobial responses because they are inexpensive and easy to perform. However, experimental factors that influence assay measurements, especially factors that affect antifungal drug tolerance, have not been standardized; antifungal drug tolerance is defined as the ability of a subpopulation of cells to grow slowly at drug concentrations that inhibit growth of the remaining population. Here, we examined how modifying details of the drug format, media properties, and the initial cell number affects the ability to measure resistance and tolerance levels. We provide specific recommendations for performing reliable and robust disk diffusion assays that report both drug susceptibility and tolerance levels.

## INTRODUCTION

Fungal pathogens infect over a billion people annually, with over 1 million deaths per year from invasive candidiasis (1, 2). Infections with *Candida* species are highly prevalent, with *Candida albicans* accounting for >50% of all candidal infections and having mortality rates ranging from 20% to 50% (3, 4). Resistance is usually due to mutations that directly affect drug/host-target interactions (5). Yet, the level of drug resistance amongst *C. albicans* clinical isolates (~3%) cannot explain the 30%–50% treatment failure rates observed for systemic infections (1).

Antifungal drug susceptibility testing quantifies *in vitro* levels of susceptibility. Susceptibility is usually measured after 24 h of growth as the minimal inhibitory concentration (MIC) of the drug, with clinical breakpoints set for many species/drug pairs. Antifungal drug tolerance, also called “trailing growth,” is the ability of some cells in an isogenic susceptible population to grow slowly in drug concentrations that inhibit the growth of the rest of the population (6). Tolerance occurs in cells that test as susceptible and relies upon stress response pathways that ultimately enable slow growth despite the presence of drug concentrations that are generally inhibitory to susceptible, non-tolerant cells (6–9). Tolerance may explain a significant proportion of *C. albicans* treatment failures that occur with fluconazole (FLC) treatment of azole-susceptible bloodstream infections (6, 10).

Because tolerant cells require longer times to initiate growth, tolerance is usually measured 48 h after cells are plated in the presence of drug. Tolerance is calculated relative to susceptibility and measured as the proportion of growth that appears above the MIC after 48 h, relative to the amount of growth in the absence of drug (6). Tolerance can be estimated from both liquid and agar-based clinical assays, yet this is rarely done quantitatively and is generally not included in clinical protocols.

Susceptibility can be measured from growth in the presence of drug using liquid or agar-based assays. In liquid broth microdilution assays (BMDAs), pre-prepared microtiter plates containing a drug dilution series and media can be purchased for use in clinical labs (11). Importantly, in liquid assays the level of resistance (growth above the susceptibility breakpoint) can be overestimated if a small number of resilient cells divide more rapidly than others, outcompeting susceptible cells in the liquid culture. This type of “heteroresistance” arises occasionally and is more readily distinguished from bona fide resistance using agar-based assays, for example, disk diffusion assays (DDAs) or Epsilometer (Etest) strips, where heteroresistant subpopulations are detected as large colonies growing within the zone of inhibition (ZOI) that is generated by drug diffusing into the agar.

**Fig 1:**
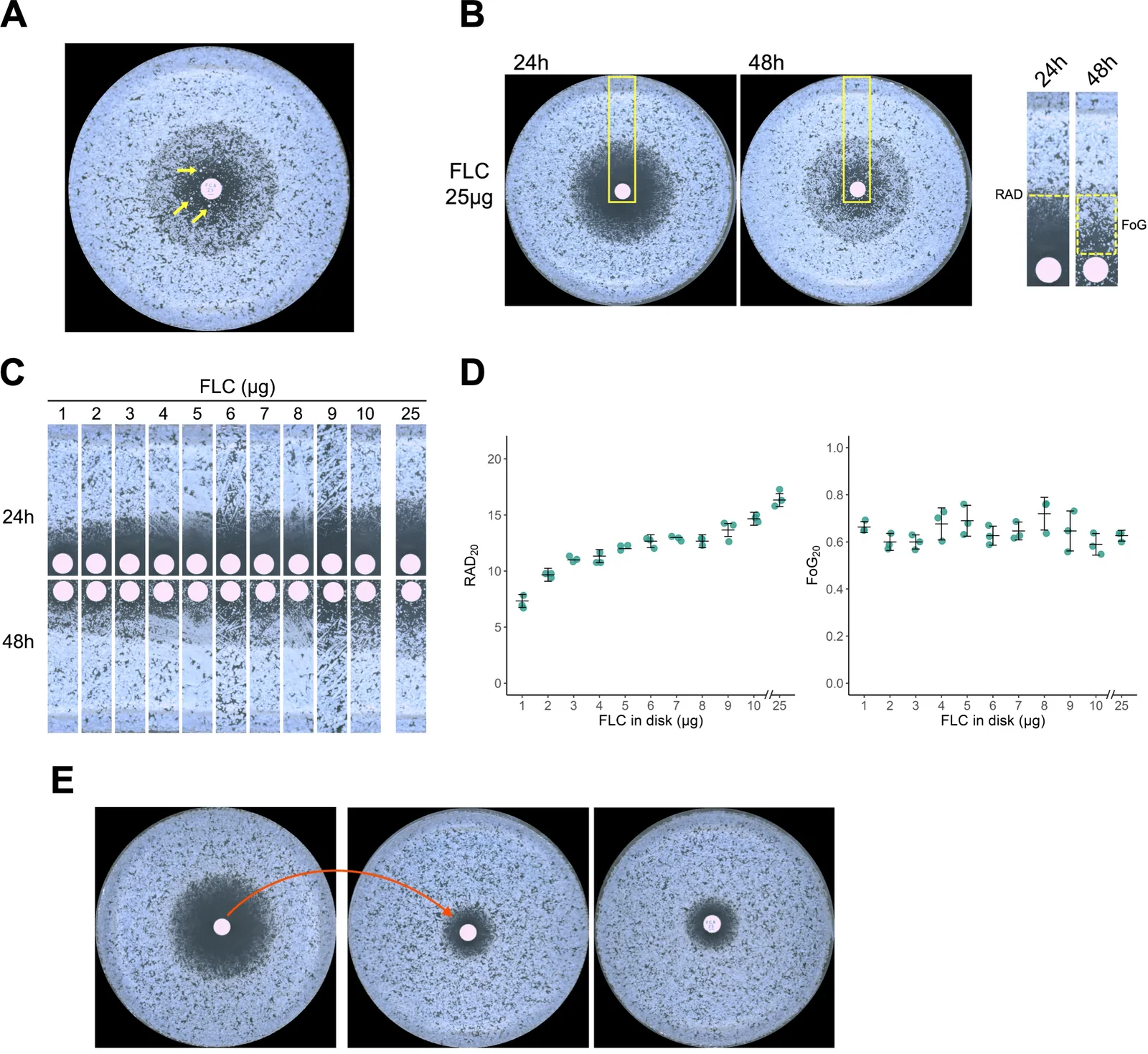
Amount of FLC per disk affects ZOI size and not tolerance levels. (**A**) Standard DDA highlighting heteroresistant colonies (arrows) detected at 48 h along with growth of abundant, smaller tolerant colonies growing inside the ZOI. (**B**) DDA plate at 24 h measures susceptibility (line, position of RAD) and at 48 h measures tolerance (rectangle, FoG). Higher RAD values indicate more susceptibility (less resistance); higher FoG measurements indicate more tolerance. (**C**) Strips from DDAs photographed at 24 h (top) and 48 h (bottom), using disks with fluconazole amounts indicated. (**D**) Susceptibility (RAD_20_ 24 h) and tolerance (FoG_20_ 48 h) data from *diskImageR* analysis of strain SC5314; *n* = 3 biological repeats per time point (RAD_20_: Spearman correlation test, *r* = 0.96, *P* < 0.001; FoG_20_: Spearman correlation test, *r* = −0.05, *P* = 0.77). (**E**) Test for amount of FLC retained in disk. Left, standard DDA with 25 µg FLC disk (RAD_20_ = 15.7 ± 0.5, *n* = 4). Right, after 24 h, the disk was removed and placed on a new standard DDA with a fresh lawn of cells (arrow). Growth on the new plate was recorded after 24 h with the pre-used disk (RAD_20_ = 5.5 ± 0.5, *n* = 4). The small RAD_20_ on this plate is comparable to the RAD_20_ seen with <1 µg in the disk (**D**).

For disk diffusion assays, diffusion of the drug from the disk produces a ZOI whose radius is inversely proportional to the MIC (12). Disk diffusion assays are cost-effective, relatively simple to perform, and have been used for ~80 years to test bacterial susceptibility to antimicrobial agents (13). In the late 1970s, DDAs were adapted to test fungal pathogens as well (14, 15). Importantly, DDAs can readily distinguish tolerance from susceptibility: susceptibility is determined by the size of the ZOI; tolerance is measured by quantifying the fungal growth that appears within the ZOI after 48 h (6), relative to growth without the drug. Tolerance can be quantified using image analysis pipelines such as *diskImageR* that calculates tolerance as the proportion of growth per unit area within the ZOI relative to the growth outside the ZOI (16), or J-AST, a new image analysis package that quantifies the growth within the ZOI for DDAs or Etests (17, 18). Heteroresistance is distinguishable from tolerance because it appears as larger, but less frequent, colonies relative to the smaller, more frequent colonies that grow from tolerant isolates (Fig. 1A). Heteroresistance is also unstably heritable, often being due to aneuploid chromosomes that are acquired and lost with some frequency (19, 20).

DDA protocols for testing fungal pathogens have been standardized by the Clinical and Laboratory Standards Institute (CLSI), and are designed to guide the selection of antifungal drugs that optimize treatment outcomes (21). The guidelines include inoculum turbidity (0.5 McFarland), medium type (Mueller-Hinton + methylene blue), pH, agar depth, as well as handling protocols to ensure consistency. Specifically, the growth medium and assay conditions aim to minimize growth ambiguity such as tolerance/trailing growth (22, 23).

Tolerance likely reflects the physiological state of the pathogen and is consequently influenced by growth conditions used to assay drug responses. Notably, initial isolates from patients with infections that did not respond well to azole treatment had higher tolerance levels than isolates causing infections that were cleared upon azole treatment (6). In addition, higher levels of tolerance were associated with high patient mortality for infections treated within 24 h of diagnosis (24). Furthermore, in animal models, high trailing growth levels were associated with poor animal survival in *C. tropicalis* infections (25). Larger-scale clinical studies are needed to determine the effect of tolerance levels on therapeutic outcomes and, perhaps, to refine clinical assays to accurately detect them. To conduct such studies, a standardized protocol for measuring antifungal drug tolerance is needed.

Research lab studies of fungal-antifungal drug interactions often use less stringent DDA protocols, which have not been standardized between labs. Here, we asked how different aspects of DDA protocols affect the reproducibility of susceptibility and tolerance measurements. Specifically, we compared resistance and tolerance measurements using different methods of drug administration, media volume, agar concentration, nutrient density, inoculum properties, and initial cell density. We highlight the protocol parameters that affected FLC tolerance and/or resistance measurements and recommend a protocol that measures them reliably.

## RESULTS

### Effect of drug concentration and format on disk diffusion assay outcomes

Standard clinical DDAs use commercial drug disks; for tests of *C. albicans,* disks usually contain 25 µg fluconazole. We first tested DDAs using 1–25 µg FLC per disk on a lawn of *C. albicans,* using our “standard” conditions (Table 1). We measured susceptibility as the radius of the ZOI at 20% inhibition (RAD_20_) at 24 h, and measured tolerance as the Fraction of Growth (FoG_20_) within the RAD_20_ (Fig. 1B and C) (16, 22). To compare RAD values to MIC values, a standard curve can be generated using strains with known MIC values determined from broth microdilution assays or Etests (16). As expected, RAD_20_ increased with FLC concentration (Spearman correlation test, *r* = 0.96, *P* = 0.001), while FoG_20_ did not (Spearman correlation test, *r* = −0.05, *P* = 0.77; Fig. 1C and D); this is consistent with the idea that tolerance is much less concentration-dependent than susceptibility/resistance (6).

**TABLE 1:** Standard disk diffusion assay conditions used

Parameters	Conditions
Drug format	Commercial disk or in-house prepared filter disk
Media	15mL in a 90mm plate^*a*^
Cell inoculum	Colony (1–4 days old).Normalize concentration to spread ~ 1 × 10^5^ per plate

^*a*^ Media: YPD (2% peptone, 2% glucose, 2% agar, and 1% yeast extract).

To establish the reproducibility of the assay, we performed technical replicates (assays of the same colony on the same day) and biological replicates (assays of different colonies on the same day and on different days) with lab strain SC5314. The average RAD_20_ value for these 27 assays was 15.8 ± SD 0.4, which translates to ±~1 mm (Table S1). For FoG_20_, the average value was 0.62 ± SD 0.036, which translates to ±0.07 units. Note that while RAD_20_ translates to mm, FoG_20_ is a relative measure of growth. Thus, differences in RAD_20_ between two strains or conditions are meaningful if they differ by >1 mm from one another, and differences in FoG_20_ if they differ by >0.07 units.

To test the effect of FLC drug format/application, we compared drug disks to direct application of a liquid droplet to the plate, liquid applied directly to the disk on the plate vs liquid dried in the disk prior to placing it on the plate, and the difference between commercial disks or in-house-prepared FLC disks with different solvents (DMSO, DDW, and ethanol) and with different solvent volumes, always using 25 µg FLC per assay (Table S2). Importantly, to the first approximation, the FLC drug format did not affect either RAD_20_ or FoG_20_ levels (*P* > 0.1, Table S2). We note that there was a small difference in the average FoG_20_ between commercial and in-house assays (*P* < 0.01); this is based on a difference of 0.03 FoG_20_ units, which is smaller than the normal variability differences within most conditions. Thus, DDAs with commercial FLC disks appear to be slightly more reproducible than assays using other FLC formats, but the difference in RAD_20_ and FoG_20_ measurements remains very small. We suggest that, whatever the chosen method, comparisons of susceptibility and tolerance are best performed using assays with the same drug format.

We next asked if the disks retain FLC after 24h by removing used disks from a DDA assay after 24 h and placing them on a new lawn of cells to perform a second DDA for 24 h. Both commercial and in-house prepared dried FLC disks had small ZOIs (RAD_20_ = 5.5 ± 0.5, Fig. 1E) corresponding to <1 µg of drug (based on RAD_20_ measurements in Fig. 1D). Thus, FLC disks retain a small amount of drug and may reduce the amount of drug that diffuses into the plate.

### Effect of media parameters on disk diffusion assay outcomes

**Fig 2:**
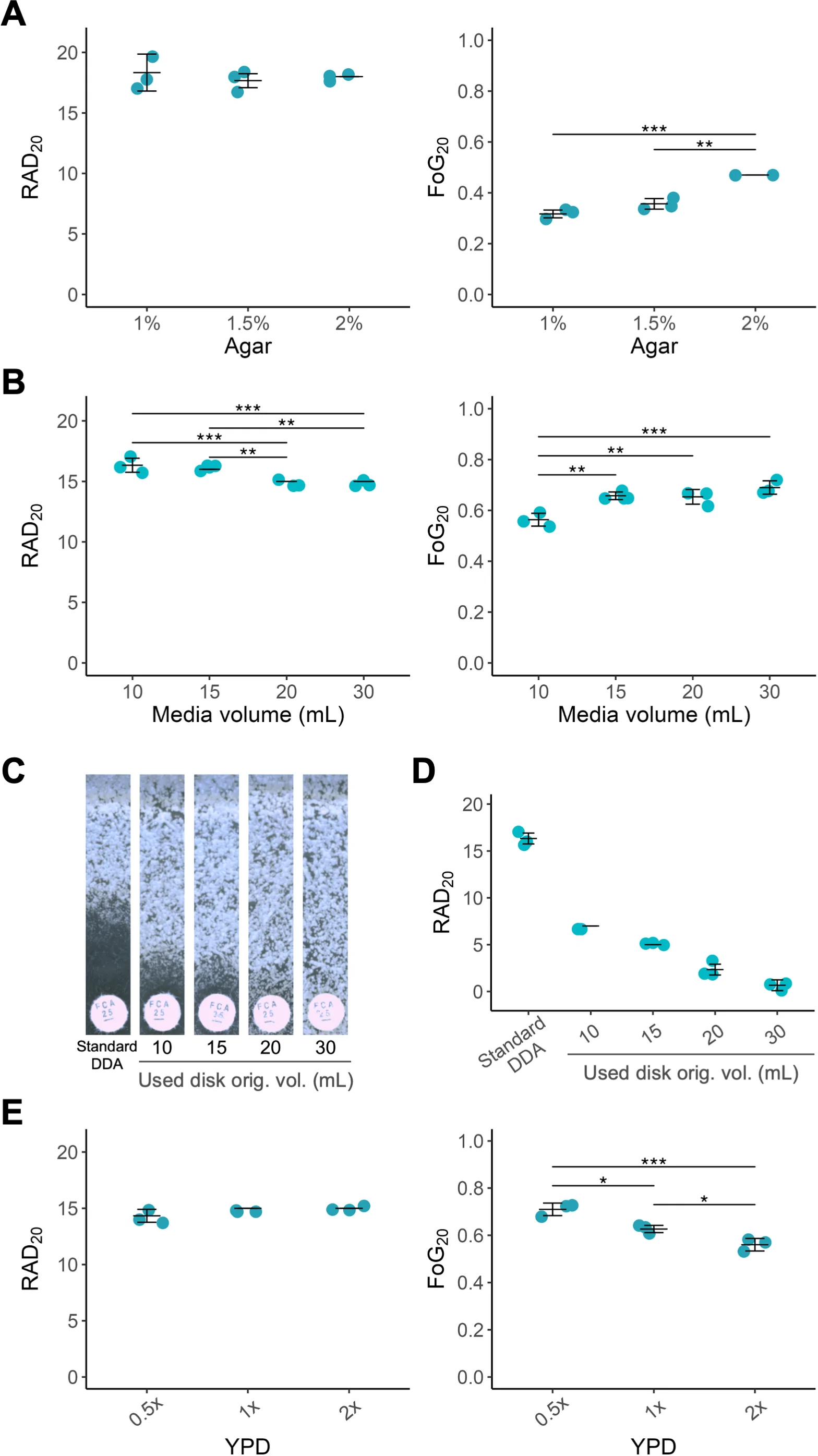
Comparison of media properties on DDA outcome. Standard DDA conditions with different agar concentrations, as indicated (**A**); or with different media volumes per plate, as indicated (**B**). (**C and D**) Used FLC disks taken from YPD plates with different volumes (10–30 mL) and placed on new standard 15 mL DDAs to assess FLC amount retained in disks. (**E**) Standard DDAs were performed on 0.5×, 1×, and 2× YPD. *n* = 3 biological replicates; comparisons found small but significant differences between some of the tested conditions (one-way ANOVA with Tukey’s post hoc test). *P < 0.05; **P < 0.01; and ***P < 0.001).

Different agar concentrations are used in different reports: CLSI guidelines suggest 1.7% agar; research reports state 1.5% or 2% agar (6, 22). DDAs performed using 1%, 1.5%, and 2% agar plates did not affect RAD_20_ levels (*P* ≥ 0.7, Fig. 2A). FoG_20_ levels did increase slightly, but significantly, with higher agar concentrations (Fig. 2A). This is consistent with tolerance measurements being more sensitive than susceptibility measurements to assay conditions. We chose 2% agar for our standard DDA conditions because it provides the best dynamic range for studying parameters that increase or decrease tolerance levels.

CLSI suggests a medium depth of 4 mm (~25.5 mL in a standard 90 mm Petri plate). DDAs performed with 10–30 mL of medium per plate found that RAD_20_ changed slightly: increased medium volume decreased RAD_20_ levels by 1–1.3 mm between all conditions (Fig. 2B). Interestingly, while FoG_20_ was lower in plates containing 10 mL of medium (0.56 units SD ± 0.02), FoG_20_ levels remained similar for plates with >10 mL medium (0.65–0.69 units, Fig. 2B). We chose 15 mL as the standard DDA conditions because there was no difference in FoG_20_ between 15 and 30 mL of media; the difference in RAD_20_ between 15 mL plates and the other volumes was ≤1 mm, and 15 mL uses fewer resources per plate than the larger volumes.

The lower RAD_20_ values at higher media volumes suggest that more media per plate dilutes the final drug concentration. Thus, we measured the amount of FLC retained in the disks (as in Fig. 1E) from plates with different media volumes (Fig. 2C and D). The RAD_20_ for the smallest volume indicated that ~1 µg of drug was retained (7 mm, SD ± 0, extrapolated from Fig. 1C) in the disk from the 10 mL media plate (Fig. 2D); increasing volumes were accompanied by even smaller RAD_20_ values, implying that less drug was retained in the disk when there was more media volume.

Some studies used diluted media (e.g., half-strength YPD) for assays of drug responses (26, 27). To ask if nutrient density alone affects RAD_20_ and FoG_20_ levels, we performed DDAs with 0.5×, 1×, or 2× YPD (Fig. 2E). RAD_20_ levels were not significantly different, while FoG_20_ levels were significantly different: highest in 0.5× YPD (0.7 SD ± 0.02), lowest in 2X YPD (0.56 SD ± 0.02), and intermediate in 1× YPD (0.62 SD ± 0.01) (Fig. 2E). This is consistent with the idea that growth conditions affect FoG_20_ levels much more than RAD_20_ levels (6).

### Effect of cell inoculum on disk diffusion assay outcomes

CLSI guidelines suggest spreading cells with a turbidity equivalent to 0.5 McFarland standard (1–5 × 10^6^ cells/mL) using a sterile swab dipped in the inoculum. In the research lab, we use 100 µL of ~10^6^ cells/mL and spread them onto medium by agitating glass beads (see Materials and Methods). We compared the effect of initial cell inoculum density using twofold dilutions ranging from ~0.25 × 10^5^ to ~8 × 10^5^ cells per assay. Initial cell density had a small but clear effect: RAD_20_ decreased linearly with increasing cell inoculum (*R*^2^ = 0.89, *P* < 0.001), and FoG_20_ increased linearly (*R*^2^ = 0.81, *P* < 0.001) with increasing inoculum. These results were consistent across seven other diverse *C. albicans* strains with different levels of RAD_20_ (*R*^2^ = 0.91, 0.91, 0.94, 0.92, 0.9, 0.82, and 0.8; all *P* < 0.001; Fig. S1) and FoG_20_ (*R*^2^ = 0.77, 0.7, 0.82, 0.91, 0.74, 0.85, and 0.94; all *P* < 0.001; Fig. S1). Thus, cell density does affect measurements of drug responsiveness within six concentration doublings.

**Fig 3:**
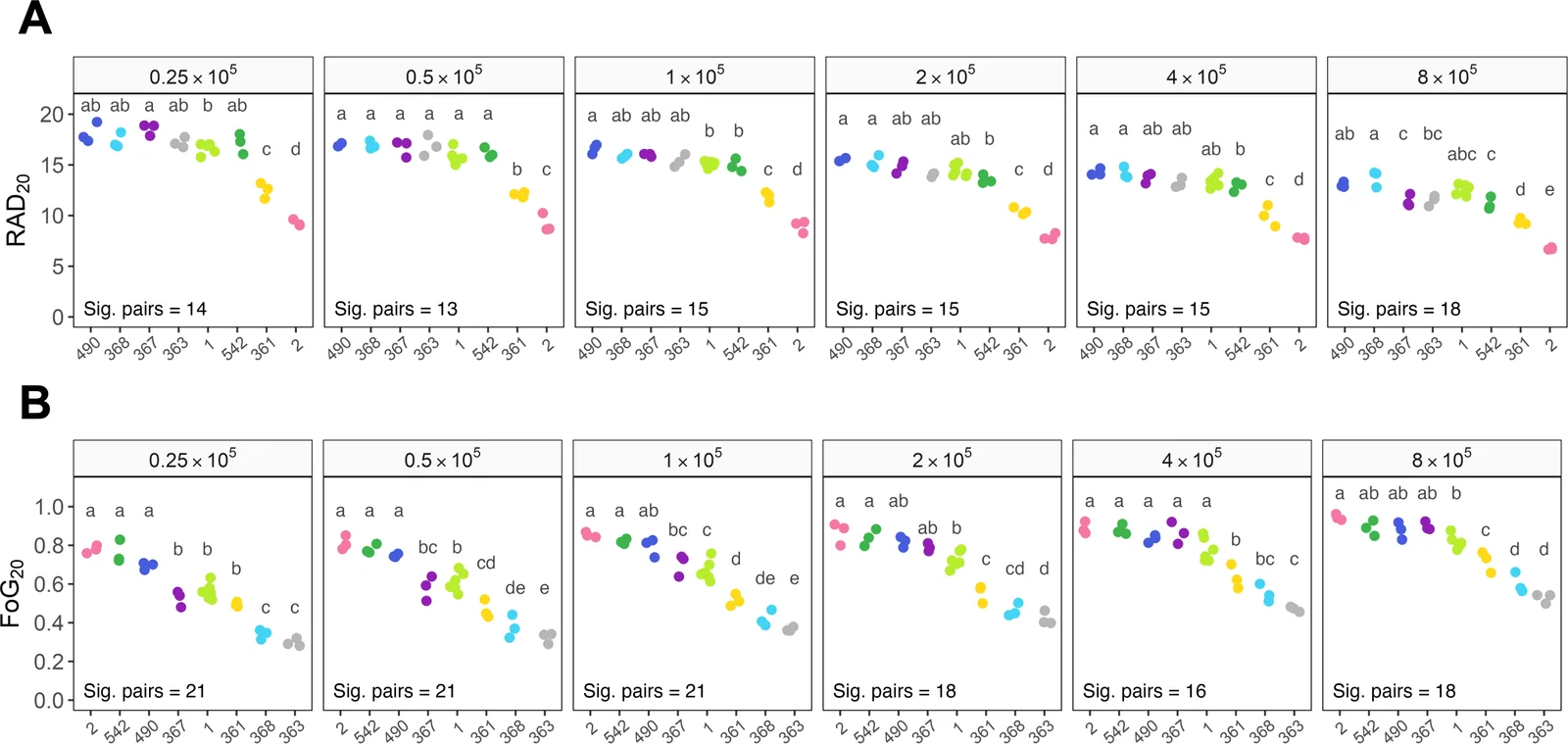
Effect of cell density on DDA outcomes across isolates. DDAs for eight strains (*x*-axis) were performed with six different cell densities as indicated (~number of cells per assay) and analyzed by *diskImageR*. (**A**) RAD_20_ measured at 24 h. (**B**) FoG_20_ measured at 48 h. Letters above data points indicate statistically significant differences between strains at a given cell density (*P* < 0.05), determined by one-way ANOVA with Tukey’s post hoc test; groups sharing the same letters are not significantly different (*P* > 0.05). *n* = 3 biological replicates.

Because DDAs are used to study the drug response properties in different mutants or strain backgrounds, we asked if they can distinguish drug responses between the eight different strains. For RAD_20_, 15 pairs of strains were distinguishable with an inoculum of ~1 × 10^5^ to ~4 × 10^5^ cells per plate (*P* < 0.05), and 18 pairs were distinguishable for 8 × 10^5^ cells per plate (*P* < 0.05). For lower inocula (~0.25 × 10^5^ or ~0.5 × 10^5^ cells per plate), the DDAs distinguished RAD_20_ values for fewer strain pairs (Fig. 3A). Thus, higher inoculum per plate increased the ability to distinguish RAD_20_ values. For FoG_20_, we saw the opposite effect, with 21 strain pairs having significant differences for ~0.25–1 × 10^5^ cells per plate (*P* < 0.05, Fig. 3B). At higher cell densities (2–8 × 10^5^ cells per plate), the number of significantly different strain pairs was lower (16–18 pairs, *P* < 0.05).

Taken together, these data suggest that using a cell density of ~1 × 10^5^ cells per assay is ideal for distinguishing different RAD_20_ levels and FoG_20_ levels between strains. This provides sufficient cell density for visualizing a clear ZOI and limits issues of overgrowth within the ZOI that occur with higher inoculum and complicate the analysis of FoG_20_ levels.

Across the eight tested strains, decreasing the inoculum from ~1 × 10^5^ to ~0.5 × 10^5^ increased RAD_20_ by 0.3–1.6 mm and decreased FoG_20_ by 0.04–0.12 units; even higher inoculum (~2 × 10^5^) reduced the RAD_20_ by 0.6–1.6 mm and increased the FoG_20_ levels by 0.01–0.08 units. These twofold variations in the initial cell concentration yielded differences in the DDA results that approach, and sometimes exceed, the threshold for RAD_20_ and FoG_20_ assay reproducibility (±1 mm, ±0.07 units, respectively). Nonetheless, because changes in initial cell concentration can affect DDA results, we suggest that normalizing the inoculum concentration will improve the ability to distinguish differences in drug responses between different strains.

We also tested other inoculum properties including the type and age of inoculum used (3-day-old vs 9-day-old colonies, Fig. S2A; log-phase vs stationary-phase liquid cultures and agar colonies, Fig. S2B; and the volume of inoculum [20–200 µL containing the same total cell number], Fig. S2C). There was no significant difference for either RAD_20_ or FoG_20_ for all but one of the conditions tested. This single comparison (inoculum from log-phase liquid culture vs from agar colonies) had a small but significant difference in RAD_20_ (1 mm, *P* < 0.05). Thus, DDAs are relatively robust to inoculum properties, and the small differences observed can be avoided by basing comparisons on DDAs performed with inoculum grown under consistent conditions.

In summary, DDAs are relatively robust to drug, media, and inoculum factors that have little, if any, effect on either RAD_20_ or FoG_20_. The only factors that consistently affect tolerance levels (albeit to a small degree) are high agar concentration, low agar volume, and nutrient density. Furthermore, for most of these parameters, if DDAs are consistently performed with the same agar concentration, volume, and nutrient density, then the *relative* RAD_20_ and FoG_20_ levels between strains will remain reliable.

## DISCUSSION

Susceptibility testing is essential for understanding fungal pathogens and their drug responses, and treatment strategies are guided by MIC levels of the relevant infecting strains. BMDAs provide precise MIC measurements; disk diffusion assays are reliable, inexpensive, easy-to-implement tests that generally yield results comparable to those of BMDAs when performed under similar growth conditions or when using a standard curve (12, 16, 28). We note that this has been reported to be problematic for azoles and *C. glabrata* (29), likely due to the 48 h time to monitor “resistance” and a lack of distinction between resistance and tolerance in strains with similar susceptibility/resistance levels. While BMDAs also can be used to monitor aspects of tolerance, by measuring “supra-MIC growth” (SMG) after 48 h (6), the relationship between the SMG parameter and FoG_20_ is not perfect because growth in liquid does not detect subpopulation effects like those that contribute to tolerance (6).

Because the CLSI guidelines for DDAs do not provide a method for preparing in-house drug disks (21), we first tested the effects of drug administration. Commercial disks with a fixed drug amount should avoid variation between disks (30), which was confirmed in DDA tests here (Table S2). FLC disks prepared in-house, regardless of the solvent and whether the FLC in the disk was dried or freshly applied to the disk on the plate, did not affect either RAD_20_ or FoG_20_. Of note, all FLC disks retained a small amount (<1 µg) of drug after 24 h on the plate. Effects on diffusion out of the disk are likely dependent on the molecular size and solubility of the drug used, which may affect drug diffusivity in the agar medium. This may be more relevant for hydrophobic and amphipathic drugs, which can interact with materials in the disk and medium; it is less of an issue for fluconazole, which is relatively small and hydrophilic (31).

Second, we investigated the effect of media by testing agar concentration, media volume, and nutrient density. Agar concentration had no significant effect on RAD_20_ values, while tolerance levels increased at high agar concentrations. The *C. albicans* lawn appeared dull and dry on 2% agar compared with 1% agar, suggesting altered cell growth and physiology. We posit that high agar concentration may affect colony hydration and nutrient diffusion (32), which, in turn, affects cell growth and the degree of tolerant growth (6).

Media volume affected FoG_20_ more than RAD_20_, suggesting that FoG_20_ may be more sensitive to the reduced amount of nutrients available in 10 mL of medium. Similarly, lower nutrient concentrations (0.5× vs 1× or 2× YPD) affected FoG_20_ but not RAD_20_. Although both conditions affected tolerance measurements, FoG_20_ was lower in 10 mL medium and higher in 0.5× YPD. This supports the idea that FoG_20_ is sensitive to many growth factors, but the specific factors involved remain to be determined. Modifying media components changes not only nutrient levels but also the osmolarity and water activity in the growth medium, which are likely to modulate the network of stress response processes that shift metabolic flux and physiological state. While we did not measure all of these responses in this limited study, the results are consistent with the idea that mechanisms of tolerance, measured as FoG_20_, depend upon stress response pathways that become activated when nutrients are more limiting (6, 10). Furthermore, because studies using limiting nutrient concentrations can detect more tolerance, we posit that measurements done under these conditions, especially if assayed at 48 h, may have been misinterpreted as reduced susceptibility/increased resistance.

Third, RAD_20_ levels between strains could be better distinguished at higher cell densities than at lower cell densities. Accordingly, cell inoculum concentration has been standardized in clinical DDA protocols (21) because it improves the ability to generate reproducible results and to distinguish the relative level of susceptibility among different strains. Unlike susceptibility, measurements of tolerance have not been standardized. Even at the lowest cell densities, FoG_20_ levels are distinguishable between different isolates, while at high cell densities, FoG_20_ may be diminished or distorted, as growth within the ZOI may be driven by the high initial cell density, rather than by *bona fide* tolerance. Thus, the dynamic range of cell density differs for measuring RAD_20_ and FoG_20_, but is generally reliable at inoculum levels of 1–2 × 10^5^ cells per plate.

In summary, disk diffusion assays clearly distinguish tolerance from susceptibility/resistance, and performing them is relatively straightforward and robust. Variation in most parameters has little effect on susceptibility and, in some cases, affects tolerance measurements. We suggest that the procedure be standardized: how the drug is administered, how agar plates are prepared (including using consistent agar concentrations and media volume), and what cell concentration is used to inoculate the plates. Details of these parameters are listed in Table 1 and are available at http://jbermanlab.com/tools.

Finally, we note that many earlier studies did not distinguish between resistance and tolerance, yet measured drug response at 48 h and in nutrient-limited media. In hindsight, the drug responses measured largely reflect tolerance rather than resistance, although this is not explicitly stated. We suggest that distinguishing between resistance and tolerance is important for gaining mechanistic insights and for making appropriate clinical decisions regarding antifungal drug treatment strategies.

## MATERIALS AND METHODS

### Disk diffusion assays

The following conditions were standard in all assays performed unless noted otherwise: Random colonies were picked from YPD agar to 1 mL of double-distilled water (DDW), and optical density (OD_620_) was measured by spectrophotometry using a Tecan Infinite F50 plate reader and adjusted to 0.03 OD_620_. Then, 100 µL (~10^5^ cells) was spread onto a 90 mm Petri plate containing 15 mL YPD agar using 5 mm glass beads. A disk containing 25 µg fluconazole (FLC) was placed in the center of the plate. All experiments were conducted with three independent random colonies. Assay parameters of the drug, media, and cells were modified according to the tested condition, as detailed below; in parallel, the same isolates were always assayed using standard DDA conditions.

### Drug

Standard DDAs were performed with a commercial 25 µg fluconazole disk (Oxoid). Alternatively, 25 µg of fluconazole (Diflucan, Combi-Blocks, 98% purity) dissolved in dimethyl sulfoxide (DMSO) was pipetted onto Whatman grade AA discs (6 mm diameter). For experiments testing the effect of drug concentration, FLC amounts were used as indicated in the figure legends. To test solvents, 25 µg FLC was dissolved in DMSO, DDW, or ethanol. To test the volume of diluent used to add drug to the disk, 2.5, 5, or 10 µL of FLC-DMSO solutions containing 10, 5, or 2.5 mg/mL, respectively, were used to add a final amount of 25 µg FLC to each disk. Disks were either placed in a sterile biohood to dry (dry disks) or used immediately (wet disks).

To test the amount of drug retained in used drug disks, commercial or in-house disks (containing 25 µg FLC) were placed on standard YPD plates and incubated at 30°C. After 24 h, the disks were lifted from these plates using sterile forceps and were placed onto plates with a lawn of new cells to perform standard DDAs at 30°C for 24 h. The same experiment was also conducted with FLC disks taken from plates with different volumes (10–30 mL); again, the used disks were removed from the plates after 24 h and tested on new standard DDAs.

### Media

YPD (2% peptone, 2% glucose, 1% yeast extract, and 2% agar) was prepared by filter sterilization of 2× YPD medium, mixed with a 2× concentration of autoclaved agar (4% agar before mixing) to yield standard 2% YPD plates. Standard YPD agar plates contained 15 mL YPD added to a 90 mm Petri dish using a sterile serological pipette. Plates were allowed to dry for 2 days at room temperature and then were stored at 4°C. To test media volume effects, plates were prepared as above, except using volumes of 10, 15, 20, and 30 mL of YPD agar per plate. To test the effect of agar concentration, plates contained 15 mL of standard YPD medium prepared with 1%, 1.5%, or 2% agar, as indicated.

To test the effect of the media nutritional density, 1X YPD plates contained 15 mL of standard YPD medium with 2% agar per 90 mm Petri dish. 0.5× YPD agar plates contained 1% peptone, 1% glucose, and 0.5% yeast extract with 2% agar, 15 mL per Petri plate; and 2× YPD medium plates contained 4% peptone, 4% glucose, and 2% yeast extract with 2% agar, 15 mL per Petri plate.

### Cells

*C. albicans* strains used are listed in Table S3. *C. albicans* cells were streaked from glycerol stocks stored at −80°C onto standard YPD agar, incubated at 30°C for 24 h, and then placed at room temperature. Colonies were randomly picked from the plate for the experiments.

For DDAs, individual young colonies that had grown for 24–96 h on YPD plates were suspended in DDW, and OD_620_ was measured to estimate cell concentration.

To test the effect of cell density, twofold dilutions of ~8 × 10^6^ cells/mL in DDW were performed five times (from ~8 × 10^6^ to ~0.25 × 10^6^ cells/mL). 100 µL of each dilution was spread per plate. To test the effect of inoculum volume (while holding cell concentration constant), cell density was estimated by measuring OD_620_, and cells were diluted with DDW accordingly. Inoculum volumes of 20–200 µL containing a fixed number of cells (~10^5^ cells per plate) were spread on plates.

To test the effect of culture growth phase, colonies were suspended in 5 mL YPD and grown overnight at 30°C with shaking at 200 rpm; to generate log-phase cells, cultures were diluted 1:50 in fresh medium and incubated at 30°C with shaking at 200 rpm for 5 h. Stationary liquid cultures were grown for at least 20 h. The log-phase and stationary-phase cultures were diluted to a standard OD_620_ (~10^6^ cells/mL) and used in otherwise standard DDAs.

To test the effect of colony age, strain SC5314 was revived from −80°C glycerol stocks by streaking onto standard YPD agar plates and incubated at 30°C for 24 h. Then, the cells were kept at room temperature for an extra 2 or 8 days. For performing DDAs, colonies were picked from the 3-day and 9-day plates and tested concurrently.

### Analysis and statistics

Disk diffusion assay images from 24 and 48 h were analyzed using *diskImageR* (16). Susceptibility was quantified after 24 h of growth as the ZOI radius at 20% inhibition (RAD_20_), and tolerance was quantified after 48 h of growth as the fraction of growth (FoG_20_) within the RAD_20_. *diskImageR* calculates the fraction of growth (FoG) within the ZOI as the pixel intensity of growth within the ZOI, normalized to the potential for growth outside the ZOI (16). All experiments were performed with three biological replicates. Spearman’s rank correlation test, two-tailed Student’s *t*-test, one-way analysis of variance, and Tukey’s test were performed for statistical analyses.

## Data Availability

All data supporting the findings of this study are presented within the manuscript as figures and tables.

## SUPPLEMENTAL MATERIAL

Supplemental tables and figures spectrum.01390-26-s0001.pdf Tables S1 to S3, and Fig. S1 and S2.

ASM does not own the copyrights to Supplemental Material that may be linked to, or accessed through, an article. The authors have granted ASM a non-exclusive, world-wide license to publish the Supplemental Material files. Please contact the corresponding author directly for reuse.
