# Supplementary material for: Parameters affecting the reliable measurement of fluconazole tolerance using disk diffusion assays with Candida albicans
Source: Microbiol Spectr. 2026 Aug 21;14(10):e01390-26. doi: 10.1128/spectrum.01390-26 (PMC13689009; doi:10.1128/spectrum.01390-26)
Supplement: Supplementary file 1 [file spectrum.01390-26-s0001.pdf]

Table S1. DDA reproducibility for lab strain SC5314

Experiment	Replicate	RAD_{20}		FoG_{20}	
		Mean	SD	Mean	SD
1	Technical (*n*=4)	15.75	0.50	0.65	0.039
2	Biological (*n*=8)	15.87	0.35	0.64	0.019
3	Biological (*n*=3)	16	0.00	0.59	0.015
4	Biological (*n*=3)	15.66	0.57	0.60	0.017
5	Biological (*n*=3)	16.33	0.57	0.57	0.049
6	Biological (*n*=6)	15.83	0.40	0.64	0.024

Table S1. DDA reproducibility for lab strain SC5314. Each DDA experiment was performed on a different day using standard conditions (Table 1). Technical replicates are assays of the same colony; biological replicates are assays of different colonies. RAD_{20} and FoG_{20} were quantified using *diskImageR*.

Table S2. Comparison of drug format used in DDAs

Fluconazole 25µg		RAD_{20} (mean±SD)	FoG_{20} (mean±SD)	*p*-value	
				RAD_{20}	FoG_{20}
**Format (in DMSO)**	Drop	16±0	0.57±0.02	0.64	0.42
	Disk (wet)	15.66±1.15	0.62±0.09		
**Volume (in DMSO, in disk)**	2.5µL (wet)	16.66±0.57	0.57±0.03	0.48	0.81
	2.5µL (dry)	16.33±0.57	0.57±0.02		
	10µL (wet)	16.33±0.57	0.58±0.05		
	10µL (dry)	16±0	0.59±0.01		
**Solvent (in disk)**	DMSO	16.33±0.57	0.67±0.04	0.54	0.51
	DDW	16.33±0.57	0.62±0.02		
	Ethanol	15.66±1.15	0.65±0.05		
**Source**	Purchased disk	16±0	0.6±0.005	0.27	0.01*
	In-house disk (in DMSO)	15.5±0.7	0.63±0.0		

Table S2. Comparison of drug format used in DDAs. In each assay, 25µg of FLC was applied to a plate (using a disk or a drop directly to the plate). Volumes of drug (as indicated) were added directly in DMSO solvent to the disk on the plate (wet) or to the disk in a dry sterile petri plate, which was then dried before placing it on the agar plate (dry). Drug solutions in sterile double-distilled water (DDW) or ethanol were compared to the standard DMSO. The source of the FLC disks was either commercial (Oxoid) or dry disks prepared as described above (in-house). Application of the drug to the plate, using a purchased disk or a 5µl liquid drop in the center of the plate (Drop), was also

compared. *n*=3 biological repeats. (*Statistical significance was determined by t-test or one-way ANOVA with Tukey's post hoc test).

Table S3. Strains used in this study. Source and strain identity.

YJBT number	Strain name	Clade	Genotype
1	SC5314	1*	Wild type
2	FH1	n.d.#	Clinical isolate MTL a/alpha
361	L26	1*	MTL a/a
363	P87	SA*	MTL a/a
367	GC75	SA*	MTL alpha/alpha
368	P78048	1*	MTL alpha/alpha
490	OKP90	2^	MTL a/a
524	AM2003/0069	2†	MTL a/alpha

* Hirakawa et al., 2015. doi:10.1101/gr.174623.114
^ MacCallum, 2009. doi:10.1128/EC.00387-08
† Lockhart et al., 2002. doi: 10.1093/genetics/162.2.737
# Marr, KA et al. 1997. doi: 10.1086/515553
n.d. not determined.

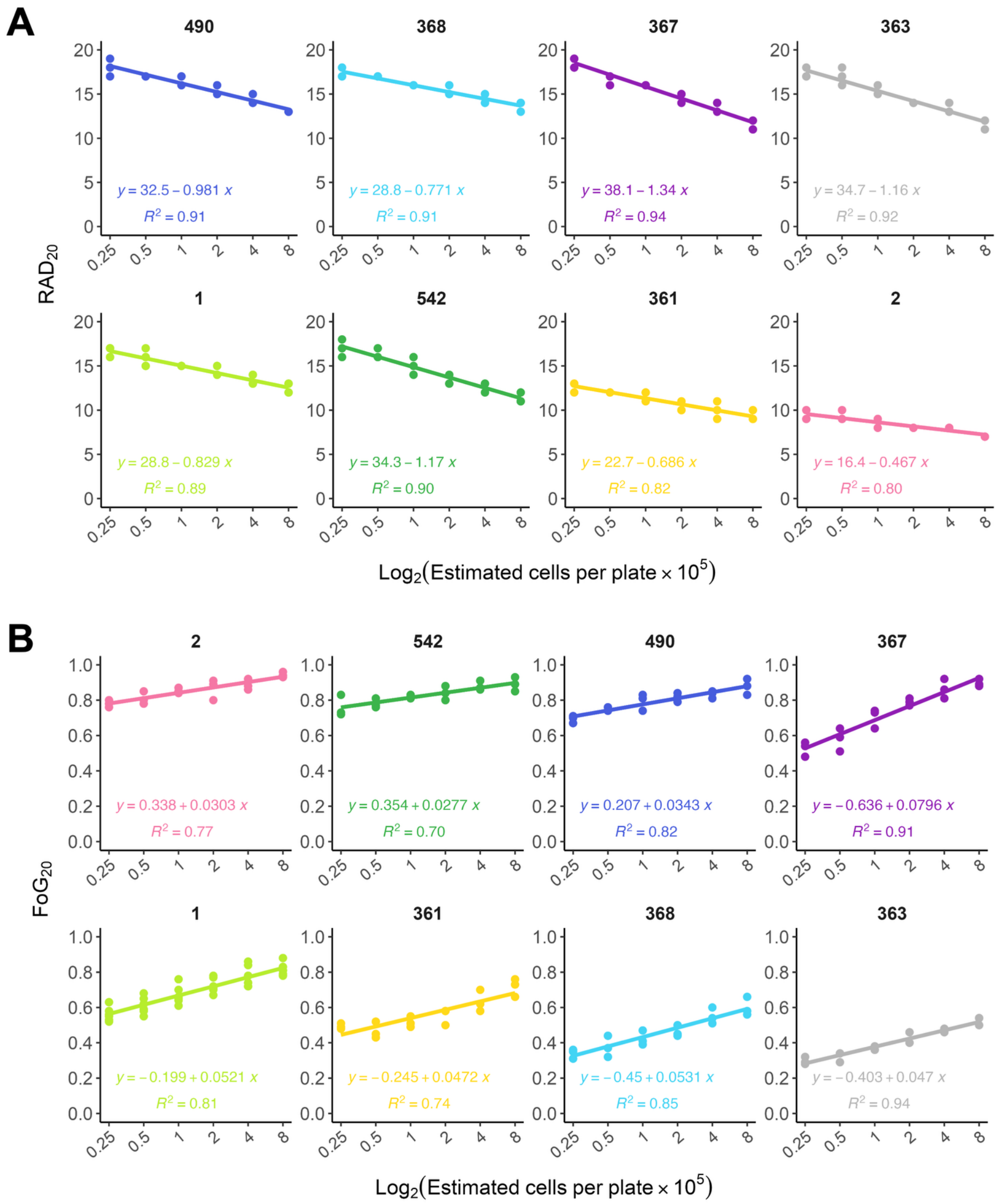

Figure S1. Associations between RAD_{20} or FoG_{20} and initial inoculum concentrations, based on experiments in Figure 3. All R^2 values and slopes obtained by linear regression analysis of RAD_{20} or FoG_{20} versus log2-transformed inoculum concentration were significant (p< 0.001). n=3 biological replicates for each strain at each inoculum concentration.

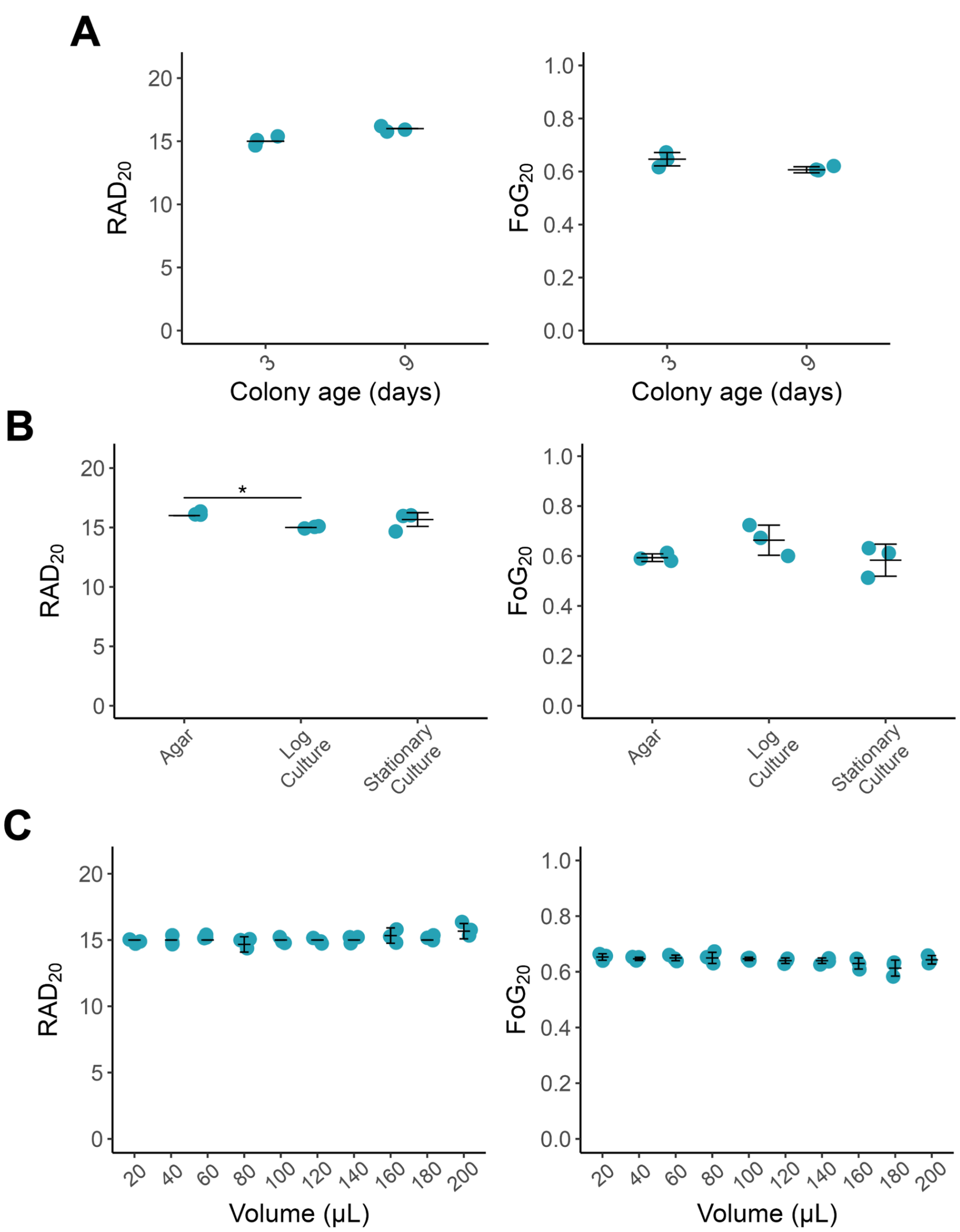

Figure S2. Comparison of inoculum properties on DDA outcome. (A) Standard DDAs performed for 3-day-old and 9-day-old colonies. RAD_{20} (24h) and FoG_{20} (48h) were quantified by *diskImageR*. **(B)** Standard DDAs performed with cells from colonies streaked on YPD agar, log phase liquid culture, or stationary phase liquid culture. **(C)** RAD_{20} (24h) and FoG_{20} (48h) for DDAs performed in standard conditions. A fixed cell concentration (~10^5 cells per plate) was spread in an inoculum of different volumes (20-200μL). *n*=3 biological replicates. (Statistical significance was determined by one-way ANOVA with Tukey's post hoc test. * $p< 0.05$).
